# Ficolin-2 Levels and *FCN2* Haplotypes Influence Hepatitis B Infection Outcome in Vietnamese Patients

**DOI:** 10.1371/journal.pone.0028113

**Published:** 2011-11-22

**Authors:** Hoang V. Tong, Nguyen L. Toan, Le H. Song, Eman Abou Ouf, C.-Thomas Bock, Peter G. Kremsner, Jürgen F. J. Kun, Velavan TP

**Affiliations:** 1 Institute of Tropical Medicine, University of Tübingen, Tübingen, Germany; 2 Department of Pathophysiology, Vietnam Military Medical University, Hanoi, Vietnam; 3 Tran Hung Dao Hospital, Hanoi, Vietnam; 4 Department of Molecular Pathology, University Hospital of Tübingen, Tübingen, Germany; 5 Robert Koch Institute, Berlin, Germany; Saint Louis University, United States of America

## Abstract

Human Ficolin-2 (L-ficolins) encoded by *FCN2* gene is a soluble serum protein that plays an important role in innate immunity and is mainly expressed in the liver. Ficolin-2 serum levels and *FCN2* single nucleotide polymorphisms were associated to several infectious diseases. We initially screened the complete *FCN2* gene in 48 healthy individuals of Vietnamese ethnicity. We genotyped a Vietnamese cohort comprising of 423 clinically classified hepatitis B virus patients and 303 controls for functional single nucleotide polymorphisms in the promoter region (-986G>A, -602G>A, -4A>G) and in exon 8 (+6424G>T) by real-time PCR and investigated the contribution of *FCN2* genotypes and haplotypes to serum Ficolin-2 levels, viral load and liver enzyme levels. Haplotypes differed significantly between patients and controls (P = 0.002) and the haplotype AGGG was found frequently in controls in comparison to patients with hepatitis B virus and hepatocellular carcinoma (P = 0.0002 and P<0.0001) conferring a protective effect. Ficolin-2 levels differed significantly between patients and controls (p<0.0001). Patients with acute hepatitis B had higher serum Ficolin-2 levels compared to other patient groups and controls.The viral load was observed to be significantly distributed among the haplotypes (P = 0.04) and the AAAG haplotype contributed to higher Ficolin-2 levels and to viral load. Four novel single nucleotide polymorphisms in introns (-941G>T, -310G>A, +2363G>A, +4882G>A) and one synonymous mutation in exon 8 (+6485G>T) was observed. Strong linkage was found between the variant -986G>A and -4A>G. The very first study on Vietnamese cohort associates both Ficolin-2 serum levels and *FCN2* haplotypes to hepatitis B virus infection and subsequent disease progression.

## Introduction

Hepatitis caused by hepatitis B virus (HBV) is one of the most serious global health problems. HBV infects more than 350 million people worldwide and remains the major cause for acute and chronic hepatitis, liver cirrhosis and for hepatocellular carcinoma (HCC). HBV related deaths constitute approx a million people worldwide [1]. In Vietnam where HBV is epidemic, individuals with chronic HBV infection were estimated around 8.4 million cases with 23,300 registered deaths in 2005 [2]. Studies have well documented that genetic and other environmental factors influences the HBV predisposition.

Genetic susceptibility to hepatitis has been investigated in *IFNA2b*, *IFNAR1*, *HLA* loci and in many interleukin related genes [3]–[6]. Our previous study reported the functional role of Mannose-binding lectin (MBL) gene polymorphisms to HBV disease outcome [7]. Similar to MBL, ficolins are pattern-recognition proteins involved in innate immunity that binds to specific pathogen-associated molecular patterns on the microbial surface and trigger the immune response either by binding to collectin receptors or by initiating the complement lectin pathway. Three ficolins were identified in humans: ficolin-1 (M-Ficolin), ficolin-2 (L-Ficolin) and ficolin-3 (H-Ficolin) and are encoded by the ficolin genes *FCN1*, *FCN2* and *FCN3* respectively [8]. The three different proteins have divergent function and are being expressed in different cells and tissues. *FCN2* gene, located on chromosome 9q34 consists of eight exons and is expressed primarily in liver cells [9]. Single nucleotide polymorphisms (SNPs) in the *FCN2* gene had been studied in different populations and was demonstrated that SNPs at positions -986G>A, -602G>A and -4A>G in the promoter region and at +6424G>T in exon 8 were significantly associated with varying serum Ficolin-2 levels and contribute towards susceptibility on many clinical infectious diseases [10]–[12].

Low Ficolin-2 serum levels and *FCN2* gene polymorphisms were associated to several infectious diseases such as respiratory infections in children and invasive pneumococcal disease in adults [13]–[15]. We had earlier investigated the possible associations between *FCN2* genotypes and haplotypes to Ficolin-2 levels in rheumatic fever, rheumatic heart disease, leprosy and in malaria [16]–[18]. Ficolin-2 is mainly expressed in the liver where hepatitis B viruses invade, replicate and finally damage liver cells. The functional role of Ficolin-2 in HBV-infections yet remains unclear and is presumed that Ficolin-2 may possibly play role in clearance of viral particles thereby protecting the liver cells from HBV infection. A recent study has reported the functional role of L-ficolins in recognition and binding of HCV envelope glycoprotein and demonstrated serum Ficolin-2 levels were associated to HCV outcome [19]. However, to the best of our knowledge, no studies had investigated the contribution of Ficolin-2 serum levels and *FCN2* gene polymorphisms in HBV outcome. In this study, we aim to demonstrate the functional role of Ficolin-2 during hepatitis and investigate any possible contribution of *FCN2* gene polymorphisms towards clinical progression of the HBV infection.

## Materials and Methods

### Ethics statement

The study was approved by the institutional review board of the Tran Hung Dao Hospital, Hanoi. Informed written consent was given by all participants.

### Patients and controls

Four hundred and twenty three (n = 423) Vietnamese HBV-infected patients were enrolled for this study. We classified the HBV patients into five different groups based on clinical, biochemical and serological diagnosis. The clinically classified groups were acute hepatitis B (AHB; n = 50), chronic hepatitis B (CHB; n = 75), liver cirrhosis (LC; n = 120), hepatocellular carcinoma (HCC; n = 123) and asymptomatic HBV carriers (ASYM; n = 55). All patients were confirmed positive for HBsAg, negative for antibody against hepatitis C virus (anti-HCV) and negative for human immunodeficiency virus (anti-HIV). Patients were clinically classified based on their prodromal symptoms, liver biochemical tests, serology for HBV and by diagnostic tests for HCC and LC as described from our previous studies [7], [20]. The patients representing asymptomatic HBV carrier group were healthy during sampling procedure, with normal liver enzyme levels and no serologic evidence for any co-infection with hepatitis C or with HIV. Neither of these individuals had a history of alcohol or drug use nor received any antiviral or immunosuppressive therapy before or during the course of this study. The characteristics such as age, gender, and clinical data such as liver biochemical tests, viral load for all 423 patients and 303 control individuals are summarized in Table 1. All patients were enrolled from 2000 to 2002 at Tran Hung Dao hospital, Bach Mai hospital, 103 Military Hospital, Hanoi, Vietnam. As for control group, we recruited 303 Vietnamese blood donors. All control individuals were confirmed negative for HBsAg, anti-HCV and anti-HIV by routine serological procedures. The serum was separated from the blood and subsequently aliquots were transferred to a fresh polypropylene tube and were stored at −70°C until use.

**Table 1:** Characteristics of Vietnamese HBV patients and control individuals.

Characteristics	AHB (n = 50)	ASYM (n = 55)	CHB (n = 75)	LC (n = 120)	HCC (n = 123)	Healthy controls (n = 303)
Age (years)	35 [17–70]	46 [21–57]	40 [19–78]	50.5 [17–78]	52 [15–77]	36 [19–48]
Gender (M/F)	39/11	46/9	56/19	98/22	98/25	215/88
ALT* (IU/l)	923 [115.5–4593]	<30	122 [11.8–2637]	48 [9–591]	43 [3–219]	<30
AST* (IU/l)	867 [182–4425]	<30	137 [17.2–1782]	74.5 [12–720]	56 [16–513]	<30
Total bilirubin* (mg/dl)	129.75 [15–558]	<17	38.05 [4.14–788]	28.3 [1.2–752]	14.1 [1.9–290]	<17
Direct bilirubin* (mg/dl)	109.8 [5.75–512]	ND	16.9 [2.5–472]	15.75 [0.9–450]	6.7 [1–212]	NA
Prothrombin* (% of standard)	88 [25–120]	>90	68 [38–100]	47 [20–100]	70 [25–100]	>90
HBV Viral load* (copies/ml)	13092[3502–4.99x10^6^]	23506 [1260–3.78x10^6^]	26719 [543–4.04x10^6^]	4620 [210–2.34x10^6^]	12449 [410–3.14x10^6^]	NA
Alpha-feto protein (AFP)* (mg/L)	NA	NA	NA	9.29 [1.2–1050]	77.77 [1.26–3260]	NA

AHB =  acute hepatitis B; CHB =  chronic hepatitis B; LC =  liver cirrhosis; HCC =  hepatocellular carcinoma;

AST and ALT =  aspartate and alanine amino transferase; IU =  international units; NA =  Not available. Values given are medians.

*P<0.001 significantly different among patient groups.

### DNA extraction

Genomic DNA was isolated from peripheral whole blood samples obtained from patients and control individuals using the commercially available QIAamp Blood mini kit (Qiagen GmbH, Hilden, Germany) following the manufacturer's instructions.

### *FCN2* genotyping

The promoter and coding regions of the *FCN2* gene (exon 1 to exon 8) were sequenced in 48 control individuals. PCR amplifications were carried out in 25 µl volume of reaction mixture containing 1X PCR buffer (20 mM Tris pH 8.8, 10 mM KCl, 1.5 mM MgCl2 and 0.1% Triton X-100), 0.2 mM dNTPs, 1 mM MgCl2, 0.15 µM of each primer, 1 unit of Taq polymerase (Qiagen) and 50 ng of genomic DNA. The cycling conditions were performed as following: denaturation at 95°C for 5 minutes followed by 40 cycles of 94°C for 30 sec denaturation, temperature specific annealing for 30 sec (56°C for promoter and exon1, exon4+6, exon7+8; 64°C for exon2+3), followed by an extension at 72°C for 45 sec and a final extension of 72°C for 5 minutes. All the PCR products were purified by using PCR DNA Purification Kit (GE Healthcare Europe GmbH) and 1 µl of the purified product were directly used as templates for sequencing, using the BigDye terminator v. 2.0 cycle sequencing kit (Applied Biosystems, USA) on an ABI 3100 DNA sequencer, according to the manufacturer's instructions. DNA polymorphisms were identified when assembled with the reference sequence of *FCN2* gene obtained from NCBI database [http://www.ncbi.nlm.nih.gov/] using the BioEdit http://www.mbio.ncsu.edu/BioEdit/bioedit.html] and Vector NTI (Invitrogen) program and were reconfirmed visually from their respective electropherograms. The primers used for amplification and sequencing are listed in Table 2.

**Table 2:** Primers and probes used for genotyping and sequencing.

Position/Fragment	Primers/Probes	Primer and probe sequences
-986G>A (rs3124952)	Forward	5′-GGGTCACAGTTTAAAATCCTTCTACT-3′
	Reverse	5′-CGTATACCTAAAGCCCCCAGA-3′
	Anchor	5′-CCTCCCACTACCACCACCGCACCC--FL
	Sensor	CY5-GCCACCTGCCGCCATCG--PH
-602G>A (rs3124953)	Forward	5′-CAAGGTCTCCCCTTCAGATG-3′
	Reverse	5′-CATGAGCAGACTTGGGACT-3′
	Sensor	5′-CCTCCTGTTCATGTGCCCC--FL
	Anchor	CY5-GTGCTCTACATACTGCCCCAGGAAACAG--PH
-4A>G (rs17514136)	Forward	5′-GGAAGCGGCTGTCACTC-3′
	Reverse	5′-CCCTTACCTGGACAGGTGT-3′
	Sensor	5′-AGCAAAGACCAGAAGAGATGGA--FL
	Anchor	CY5-CTGGACAGAGCTGTGGGGGTC--PH
+6424G>T(Ala258Ser) (rs7851696)	Forward	5′-TGCCTGTAACGATGCTCA-3′
	Reverse	5′-TGTATCCTTTCCCCGACTT-3′
	Sensor	5′-GAAACATCACAGCACAATTTCC--FL
	Anchor	CY5-GTGTTAAGATCATTGTCCTGGTCTTTGGT--PH
Promoter and Exon 1	Forward	5′-ATT GAA GGA AAA TCC GAT GGG-3′
	Reverse	5′-GAA GCC ACC AAT CAC GAA G-3′
Exon 2+3	Forward	5′-AGA TGG CAG ATG CCT TTC AG-3′
	Reverse	5′-GTT CCT CTG CAG CCA GGT C-3′
Exon 4+6	Forward	5′-AGG CCC AGA AAA TGG TGT C-3′
	Reverse	5′-AGG CTC TTG TGT TCC AGG C-3′
Exon 5	Forward	5′-ATA CAG ACG CCT ATG GCC C-3′ ^(*)^
Exon 7+8	Forward	5′-CCA GCT CCC ATG TCT AAA GG-3′
	Reverse	5′-TTA CAA ACC GTA GGG CCA AG-3′

FL: Fluorescein; Cy5: Cyanine 5.18; PH: Phosphate group; ^(*)^: sequencing primers

For the patients and controls cohort, three SNPs including -986G>A (rs3124952), -602G>A (rs3124953), and -4A>G (rs17514136) in the promoter region and one SNP, +6424G>T (rs7851696) in exon 8 were genotyped using real-time polymerase chain reaction (RT- PCR) based on the allelic discrimination principle and fluorescence resonance energy transfer (FRET). The HybProbe including anchor and sensor probes were labeled with either fluorescein or cyanine dye. The SNP specific sensor probes were designed one nucleotide apart from the anchor probe to facilitate the energy transfer between the two fluorescent dyes (reporter and quencher) in proximity. In the melting phase, gradual increase in temperature decreases the fluorescence intensity as one of the probes melt off leaving the two fluorescent dyes apart. The sensor probe with a clear match with target DNA melts off at a higher temperature contrary to the mismatch that melts off at a lower melting temperature. Therefore, the difference in the melting temperature remains as a basis to differentiate the genotypes. Primers and probes used in this study were summarized in Table 2. In brief: an asymmetrical PCR was carried out as a two step procedure in the Rotor Gene 3000 (Corbett, Sydney, Australia). The PCR was performed in a 20 µl reaction volume containing 50ng of genomic DNA, 1X of QuantiTect Multiplex PCR NoRox Master Mix (Qiagen, Hilden, Germany), 0.2 µM of the hybridization probes, 0.05 µM of the primer (either forward or reverse) and 0.5 µM of (either reverse or forward). The thermal cycling parameters for first step: an initial denaturation at 95°C for 15 min, followed by 40 cycles of denaturation at 94°C for 1 min followed by annealing and extension at 60°C for 1 min. Subsequently for the second step: denaturation at 95°C for 1 min, cooling to 40°C for 1 min, increasing the temperature up to 90°C by rising each 1 degree and hold for 2 seconds with a final cooling to 40°C for 60 sec. A negative and positive control was included for all PCR. All genotypes were validated by their melting temperature (Tm) of the probes using the Rotor-Gene ver.6.1.81 Software (Corbett, Sydney, Australia).

### Ficolin-2 ELISA

The Ficolin-2 serum levels were measured in sera from patient groups (AHB, ASYM, CHB, HCC, and LC) and in controls. Ficolin-2 serum levels were measured in the serum using the human Ficolin-2 ELISA kit (Hycult Biotech, Uden, Netherlands) following manufacturer's instructions.

### Statistical analysis

Data had been analysed by StatView (http://www.statview.com) and the level of significance was set to P<0.05. One way ANOVA and Kruskal-Wallis tests were employed to analyze the association between serum Ficolin-2 levels and genotypes, haplotype in different clinical groups. Chi-square and Fisher exact tests were executed to determine the differences in allele frequencies and genotype distributions in clinical sub groups. Genotype or haplotype frequencies were determined by simple gene counting and by using the expectation-maximum (EM) algorithm. The significance of deviations from Hardy-Weinberg equilibrium was tested using the random-permutation procedure as implemented in the Arlequin v. 3.5.1.2 software. (http://lgb.unige.ch/arlequin). Linkage disequilibrium (LD) analysis was performed using Haploview v. 3.2 program.

## Results

### Characterization of *FCN2* gene variants

**Figure 1:**
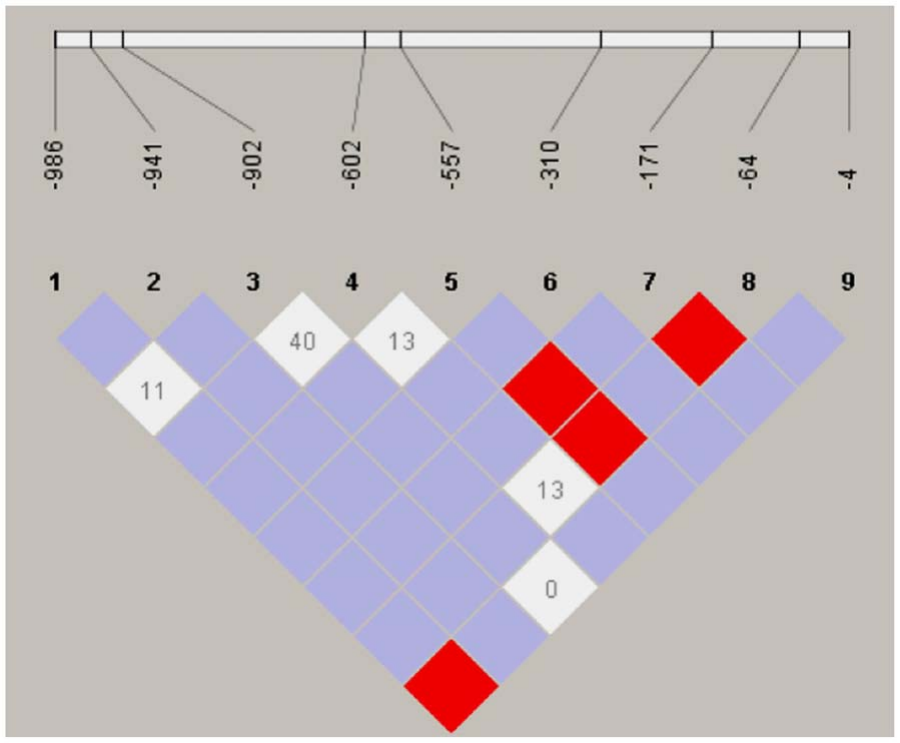
Haploview plot illustrating the linkage disequilibrium of the *FCN2* promoter region in Vietnamese individuals. At the top, the SNPs are shown according to their succession from the start of the translation of the *FCN2* gene. Open squares indicate a high degree of LD (LD coefficient D' = 1) between pairs of markers. Numbers indicate the D' value expressed as a percentile. Red squares indicate pairs in strong LD with LOD scores LD ≥3; pink squares, D' = 1 with LOD ≤2; white squares, D'<1 with LOD ≤1.

Initial characterization of 48 Vietnamese controls revealed four novel single nucleotide polymorphisms (-941G>T, -310G>A in the promoter, +2363G>A, +4882G>A in the introns) and one synonymous mutation (+6485G>T in exon 8) was observed Table 3. Linkage disequilibrium analysis revealed strong allelic combinations at positions -986G>A and -4A>G, -557A>G and -171C>T, -557A>G and -64A>C as well as in -171C>T and -64A>C (Figure 1).

**Table 3:** Distribution of *FCN2* gene variants in Vietnamese individuals (n = 48).

Position	Major allele	Minor allele	dbSNP	Region	Amino acid change	Minor allele frequency
-986	G	A	rs3124952	Promoter		0.08
-941	G	T	ss341914071	Promoter		0.01
-902	C	A	rs3811143	Promoter		0.08
-602	G	A	rs3124953	Promoter		0.02
-557	A	G	rs3811140	Promoter		0.26
-310	G	A	ss341914072	Promoter		0.01
-171	C	T	rs3811139	Promoter		0.07
-64	A	C	rs28969369	Promoter		0.26
-4	A	G	rs17514136	Promoter		0.06
+1878	T	C	rs3124955	Intron 2		0.31
+2035	T	C	rs77862660	Intron 2		0.09
+2051	T	C	rs73565973	Intron 2		0.28
+2088	C	T	rs73565979	Intron 2		0.28
+2182	G	A	rs12344423	Intron 2		0.03
+2363	G	A	ss341914073	Intron 2		0.01
+2417	G	A	rs7024491	Intron 2		0.46
+2422	G	A	rs118122273	Intron 2		0.09
+2472	A	G	rs3128624	Intron 2		0.39
+2488	T	C	rs4520243	Exon 3	Arg74Arg	0.39
+2545	G	A	rs7037264	Intron 3		0.51
+4052	A	T	rs12685659	Intron 4		0.26
+4837	G	A	rs12684512	Intron 5		0.25
+4852	C	T	rs117241205	Intron 5		0.06
+4882	G	A	ss341914074	Intron 5		0.01
+5060	C	T	rs34789496	Exon 6	His181His	0.08
+6031	A	G	rs11103563	Intron 7		0.26
+6220	T	G	rs7872508	Intron 7		0.26
+6359	C	T	rs17549193	Exon 8	Thr236Met	0.07
+6424	G	T	rs7851696	Exon 8	Ala258Ser	0.26
+6485	G	A	ss341914075	Exon 8	Arg278Leu	0.01

### *FCN2* gene polymorphisms and HBV infection

Both genotype and allele frequencies for all analysed SNP variants (-986G>A, -602G>A, -4A>G, +6424G>T) in controls and patients were in Hardy-Weinberg equilibrium. No significant differences were observed either in genotype or in allelic distributions between patients and controls or among patients groups (P>0.05), (data not shown). Four common haplotypes -986/-602/-4/+6424 GGAG, GGAT, AAAG and AGGG were observed. The haplotypes and their respective frequencies in controls and patients were summarized in Table 4. Haplotype GGAG was observed in higher frequency followed by haplotypes GGAT, AAAG and AGGG. A significant distribution among haplotypes were observed between patients with HCC and controls (χ^2^ = 21.9; P<0.0001) and also in total HBV patients and controls (χ^2^ = 14.75, P = 0.002). The AGGG haplotype was observed significantly more in controls than in patient groups (AHB vs. Controls: OR: 0.2, 95%CI: 0.02–0.8, P = 0.02; HCC vs. Controls: OR: 0.04, 95%CI: 0.001–0.25, P<0.0001 and Patients vs. Controls: OR: 0.4, 95%CI: 0.28–0.7, P = 0.0002) Table 4.

**Table 4:** Distribution of *FCN2* haplotypes (-986/-602/-4/+6424) in hepatitis B patients and controls.

Haplotype-986/-602/-4/+6424	Cases (%)	Controls (%)	OR (95%CI)	*χ^2^*	*P*
	**AHB (n = 92)**	**Controls (n = 606)**			NS
GGAG	64 (69.6)	411 (67.8)	NA		NS
GGAT	24 (26.1)	122 (20.1)	NA		NS
AAAG	2 (2.2)	15 (2.5)	NA		NS
AGGG	2 (2.2)	58 (9.6)	**0.2 (0.02**–**0.8)**	**5.56**	**0.02**
	**ASYM (n = 108)**	**Controls (n = 606)**			NS
GGAG	74 (68.5)	411 (67.8)	NA		NS
GGAT	19 (17.6)	122 (20.1)	NA		NS
AAAG	6 (5.7)	15 (2.5)	NA		NS
AGGG	9 (8.3)	58 (9.6)	NA		NS
	**CHB (n = 133)**	**Controls (n = 606)**			NS
GGAG	96 (72.2)	411 (67.8)	NA		NS
GGAT	22 (16.5)	122 (20.1)	NA		NS
AAAG	6 (4.5)	15 (2.5)	NA		NS
AGGG	9 (6.8)	58 (9.6)	NA		NS
	**HCC (n = 224)**	**Controls (n = 606)**		**21.9**	**<0.0001**
GGAG	159 (71)	411 (67.8)	NA		NS
GGAT	56 (25)	122 (20.1)	NA		NS
AAAG	8 (3.6	15 (2.5)	NA		NS
AGGG	1 (0.4)	58 (9.6)	**0.04 (0.001**–**0.25)**		**<0.0001**
	**LC (n = 239)**	**Controls (n = 606)**			NS
GGAG	168 (70.3)	411 (67.8)	NA		NS
GGAT	51 (21.3)	122 (20.1)	NA		NS
AAAG	5 (2.1)	15 (2.5)	NA		NS
AGGG	15 (6.3)	58 (9.6)	NA		NS
	**Patients (n = 796)**	**Controls (n = 606)**		**14.75**	**0.002**
GGAG	561 (70.5)	411 (67.8)	NA		NS
GGAT	172 (21.6)	122 (20.1)	NA		NS
AAAG	27 (3.4)	15 (2.5)	NA		NS
AGGG	36 (4.5)	58 (9.6)	**0.4 (0.28**–**0.7)**	**14.02**	**0.0002**

AHB: acute hepatitis B; ASYM: asymptomatic hepatitis B carriers; CHB: chronic hepatitis B; HCC: hepatocellular carcinoma; LC: liver cirrhosis. n =  Number of chromosomes; NA: not applicable; NS: not significant.

### Ficolin-2 serum levels and HBV infection

**Figure 2:**
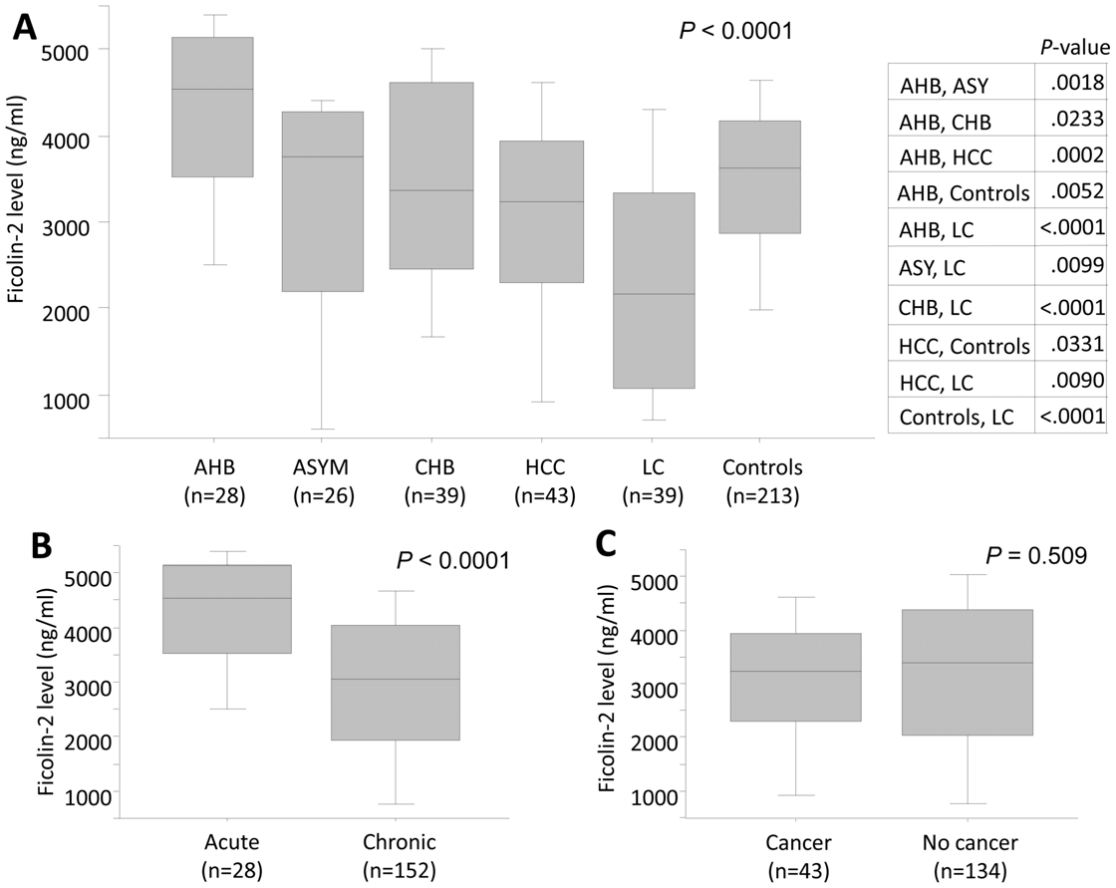
Ficolin-2 serum levels in HBV patients and controls. Box-plots illustrate medians with 25 and 75 percentiles with whiskers to 10 and 90 percentiles; *P* value was calculated by t-test. Panel (A): Ficolin-2 serum levels were segregated based on patient groups including acute hepatitis B (AHB), asymptomatic HBV carriers (ASYM), chronic hepatitis B (CHB), hepatocellular carcinoma (HCC), liver cirrhosis (LC) and controls. Statistical analysis was performed using contingency table analysis with one-way ANOVA and P-values were illustrated. Panel (B): Ficolin-2 serum levels in HBV-infected patients with acute and chronic HBV (ASYM/CHB/HCC/LC). Panel (C): Ficolin-2 serum levels in HBV-infected patients with cancer (HCC) and without cancer (AHB/ASYM/CHB/LC).

Ficolin-2 level from 213 controls varied from 0.2 to 7.0 µg/ml with a median concentration of 3.6 µg/ml. Across patients, Ficolin-2 serum levels were found with a median 5.44 µg/ml in AHB, 3.8 µg/ml in ASYM, 3.3 µg/ml in CHB, 3.2 µg/ml in HCC and 3.7 µg/ml in LC patients. Ficolin-2 serum levels differ significantly among the patient groups (P<0.0001) (Figure 2A). Ficolin-2 serum levels in AHB group were found significantly higher than others and those in LC were found significantly lower. When acute (AHB) and chronic stages (ASYM+CHB+HCC+LC) were compared, we found that the median Ficolin-2 serum levels were significantly higher in the acute stage than those in chronic group (P<0.0001) Figure 2B. In contrast, the Ficolin-2 serum levels in cancer group (HCC) and no cancer group (AHB+ASYMP+CHB+LC) were not statistically significant (P = 0.509) Figure 2C.

### *FCN2* haplotypes and serum Ficolin-2 levels

**Figure 3:**
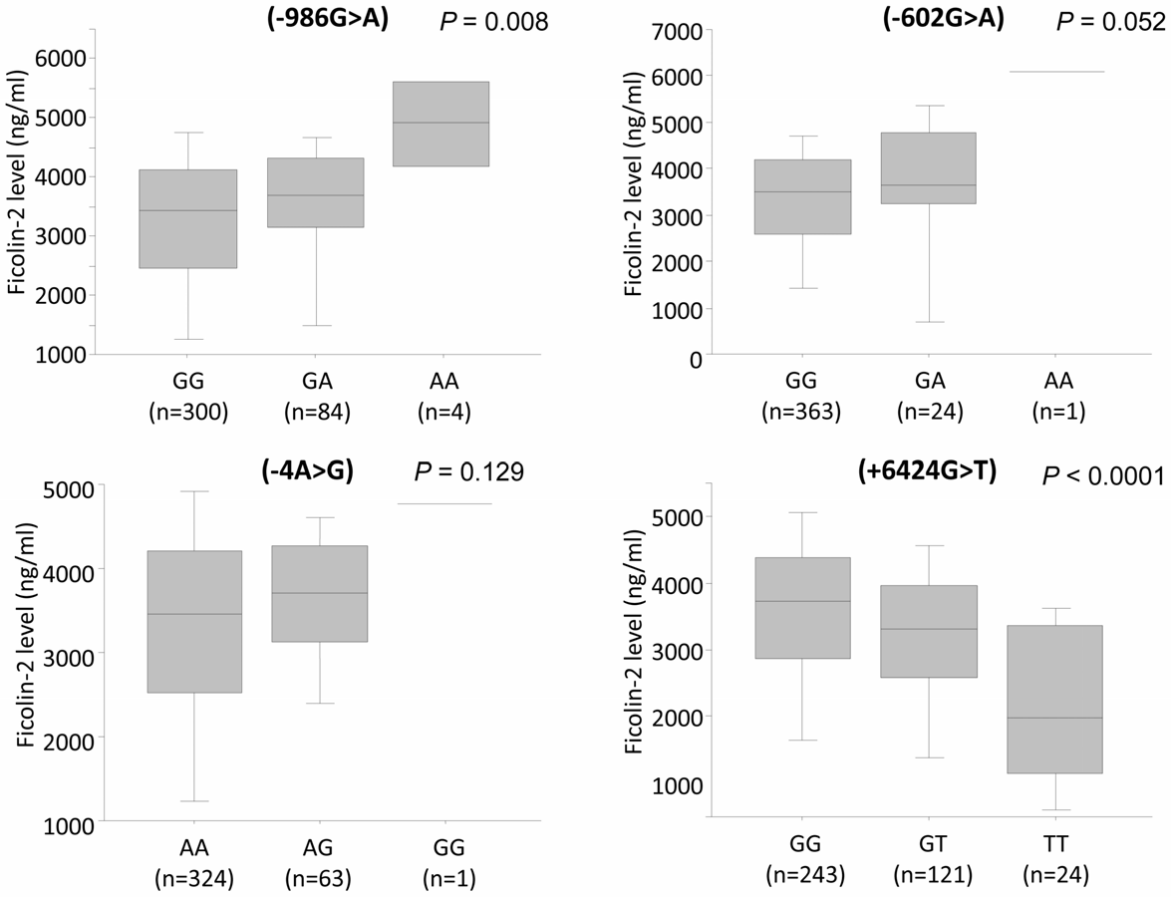
Association of Ficolin-2 serum levels and *FCN2* genotypes. Box-plots illustrate medians with 25 and 75 percentiles with whiskers to 10 and 90 percentiles; Ficolin-2 serum levels were measured and segregated based on *FCN2* SNP variants (-986G>A, -602G>A, -4A>G and +6424G>T). *P* value was calculated by ANOVA, and figures in parenthesis represent the number of samples.

**Figure 4:**
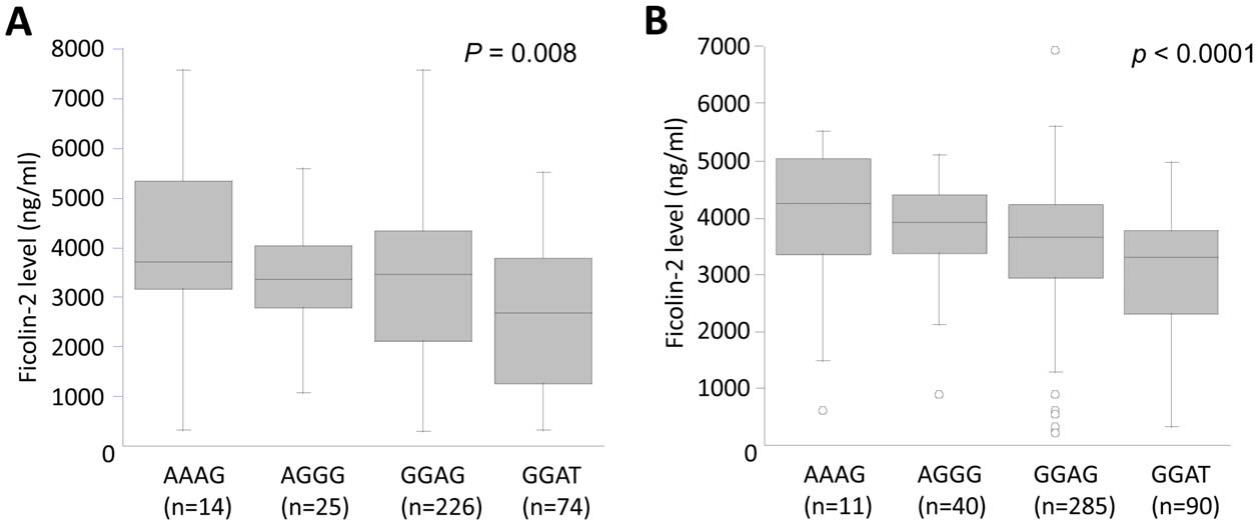
Association of Ficolin-2 serum levels and *FCN2* haplotypes. Box-plots illustrate medians with 25 and 75 percentiles with whiskers to 10 and 90 percentiles; Ficolin-2 serum level was segregated based on *FCN2* haplotypes (-986G>A, -602G>A, -4A>G and +6424G>T). Panel (A): in patients and Panel (B): in controls. The number in parenthesis indicates number of observed haplotypes. *P* values were calculated by ANOVA.

The Ficolin-2 level were significantly distributed among the three genotypes (986G>A, -602G>A, +6424 G>T) Figure 3. The minor alleles in the promoter region (-986A, -602A, -4G) contributed to an increased Ficolin-2 serum levels in contrast to the minor allele in the coding region (+6424G>T) Figure 3. The Ficolin-2 level were significantly distributed among different haplotypes in patients (P = 0.008) and in controls (P<0.0001). The AAAG haplotype had the highest Ficolin-2 levels whereas GGAT revealed lower Ficolin-2 levels Figure 4.

### *FCN2* gene polymorphisms and HBV clinical attributes

**Figure 5:**
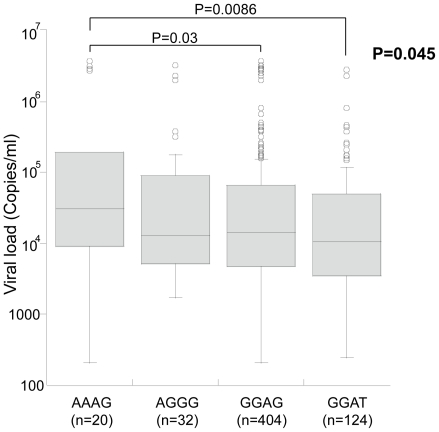
Association of viral load and *FCN2* haplotypes in HBV patients. Box-plots illustrate medians with 25 and 75 percentiles with whiskers to 10 and 90 percentiles. The number in parenthesis indicates number of observed haplotypes. *P* values were calculated by Kruskal- Wallis test.

The viral load (copies/ml) were observed to be significantly distributed among -602G>A genotypes, P = 0.04 (data not shown) and -602GA genotype was observed to have higher viral load in comparison to -602GG genotypes (P = 0.03). At the very instance, the viral load were observed to be significantly distributed among the four major haplotypes AAAG, AGGG, GGAG and GGAT, P = 0.04 Figure 5. Of which AAAG haplotype was observed to have higher viral load in comparison to other haplotypes GGAG and GGAT (P = 0.03 and P = 0.008 respectively). The level of liver enzymes such as alanine transaminase (ALT), aspartate transaminase (AST), total bilirubin (TBIL) and direct bilirubin were not significantly associated either to *FCN2* genotypes or haplotypes.

## Discussion

In this study, we evaluated the contribution of *FCN2* gene polymorphisms and the Ficolin-2 serum levels in Vietnamese HBV patients. Although several studies described associations in host genes and susceptibility to the HBV infection, none had looked in explicit on the contribution of *FCN2* gene polymorphisms. Most of the studies had focused mainly on immune system genes such as interleukins and its receptors, major histocompatibility complex (MHC) loci and human leukocyte antigen (HLA) loci to HBV infection [3], [21]. Our previous study on contribution of mannose-binding lectin (MBL) gene has demonstrated the possible association of the MBL gene variant to the HBV clinical outcome [7]. Logically, Ficolin-2, a protein similar to MBL in immunological functions, should possibly play a similar role to protect against HBV infection. When examining the contributions of genotype and allele frequencies to HBV infection, we could not find any significant difference between the patient groups and controls. Analysis at haplotype level had been shown to offer more power and further insights on multifactorial disease studies [22]. Haplotypes reconstructed based on four SNP variants (-986G>A, -602G>A, -4A>G and +6424G>T) suggested to contribute to HBV infection (χ^2^ = 14.75, P = 0.002) and a decreased risk of HCC (χ^2^ = 21.9, P<0.0001). The AGGG haplotype was found in low frequency in all HBV-infected subjects and especially in the AHB and HCC sub groups, compared to controls. These results suggest that haplotype AGGG contributes to protection against HBV infection and HCC. Nevertheless, this particular AGGG haplotypes were observed in lower frequencies in the Vietnamese population. The AAAG haplotype that was observed to be associated with higher serum Ficolin-2 levels were in lower frequency both in clinically classified patients as well as in controls Table 4. Studies have shown that polymorphisms in *FCN2* reveal different geographical patterns including Denmark, Mozambique, Ghana, Japan and Argentina [11]. The frequency of the SNP variant -986G>A significantly differ in various study groups. The variants -602G>A and -4A>G were observed in lower frequencies in African and Japanese populations respectively. In Vietnamese population, the minor allele variants -986G>A, -602G>A and -4A>G were found in frequencies 0.08, 0.02 and 0.06 respectively. Allele frequencies at positions -986G>A and -4A>G were lower than those of Caucasian and African populations. Low allele frequencies in these positions may contribute to the decreased Ficolin-2 levels in Vietnamese population (3.6 µg/ml) in comparison to Danish Caucasians (3 µg/ml) and Gabonese (11.4 µg/ml) population [11], [12], [16]. In addition, several new SNP variants in the promoter region such as -941G>T, -310G>A and new SNP variants +2363G>A, +4882G>A, +6485G>T in exon and intron regions were discovered in Vietnamese population. However the functional contribution of these novel variants is yet to be established.

Earlier studies have established that three promoter SNP variants (-986A, -602A and -4G) were associated to increased ficolin levels and the SNP variant in exon 8 (+6424T) to decreased Ficolin-2 serum levels. Our results reconfirmed that these SNP variants in the promoter region and in exon 8 contributed to varying serum Ficolin-2 levels, which were well in accordance with previous studies [10]. These results corroborate with previous studies that described *FCN2* genotypes association with varying Ficolin-2 serum levels in a gene-dependent manner [10], [12]. Serum Ficolin-2 serum levels has been shown to vary between 1 to 12 µg/ml in different populations [10], [12], [16], [23], [24]. Ficolin-2 can recognize and bind to several specific carbohydrate structures such as GlcNAc exposed on the surface of pathogens, activate the lectin complement pathway through association with MASP [9]. More importantly, one study has demonstrated that Ficolin-2 can recognize DNA elements and participate in the clearance of dying host cells [25]. Recent studies have described the binding of Ficolin-2 to N-glycans on HCV envelope activate the lectin complement pathway-mediated cytolytic activity in HCV-infected hepatocytes [19]. Hypothetically, we can expect similar interaction between Ficolin-2 and HBV antigens that can possibly trigger the lectin mediated complement pathway. However, further studies remains mandatory to confirm this interaction. Our results demonstrated that Ficolin-2 serum levels were found in higher patients with acute hepatitis B. During an acute stage, both innate and adaptive immunity are activated leading to increased production of cytokines and chemokines by cytotoxic T lymphocytes resulting in liver injury. Infected cells also can detect the presence of viral components via specific molecules and produce antiviral interferon as well as other pro-inflammatory cytokines [26]. Liver is described as an immunological organ where 80 to 90% of complement components and secreted pattern-recognition receptors of the innate immune system are synthesized [27], [28]. The complement system plays a pivotal role in determining the pathogenesis of liver diseases such as liver injury and repair, viral hepatitis and liver cirrhosis [29]. It can hold true that complement activators like MBL and Ficolin-2 also were increasingly produced to activate complement system through the lectin pathway that contribute to the clearance of virus infected cells. Moreover, HBV is believed to be a stealth virus that does not modify gene expression of the host liver during the early stage of infection and does not elicit a strong innate immune response [30]. However, our results demonstrate that Ficolin-2 serum levels remained higher in acute phase than others stages, suggesting that Ficolin-2 may play a crucial role in direct HBV clearance or by removing dying infected cells. Ficolin-2 serum levels were of the lowest concentration in patients with liver cirrhosis. However, the reason why ficolin-2 serum level in patients with liver cirrhosis is lower is yet to be elucidated. Contrary to our results with Ficolin-2 measurements, one reported study inferred that serum Ficolin-2 levels in liver cirrhosis patients caused by hepatitis C virus (HCV) were significantly higher than those in controls and other groups including chronic inactive-HCV and active-HCV patients [25]. One possible reason for decreased Ficolin- 2 levels in patients with liver cirrhosis is that during cirrhosis, the liver tissues are damaged and the blood circulation in the organ may be blocked and resulting in decreased Ficolin-2 production or a reduced secretion in the peripheral blood. The contrary results in other studies for Ficolin-2 serum level in liver cirrhosis patients may be due to the different viral factors, the sample size or different utilized tests employed in the study. Low serum mannose binding lectin (MBL) has been described to associate with occurrence of cirrhosis as well as HCC and MBL could bind to hepatitis B surface antigen (HBsAg) in a dose-dependent manner and genotypes of MBL influence recovery of HBV infection [31], [32]. When examining the correlation of viral load and Ficolin-2 serum levels, we observed that Ficolin-2 serum levels were negatively correlated to HBV-DNA viral load only in AHB group. However this correlation was weak and remained statistically insignificant (Pearson's r =  -0.266, P = 0.21) (data not shown). The plausible explanation for a non-significant trend could be due to low sample size (n = 24). When examining the correlation of viral load and Ficolin-2 haplotypes, we observed that ficolin-2 serum levels were significantly higher in AAAG haplotypes in comparison to other haplotypes. Further studies about biological functions of Ficolin-2 in hepatitis B infection need to be carried out to elucidate the associations of Ficolin-2 and HBV infection. Overall, our data suggests that Ficolin-2 not only play an important role in the pathogenesis of HBV infection but also a functional role in other liver diseases caused by HBV.

This current study provides the first insights on contribution of the *FCN2* gene polymorphisms and the Ficolin-2 serum levels to HBV outcome in a Vietnamese cohort. The reconstructed AGGG haplotype (-986G>A, -602G>A, -4A>G and +6424G>T) were observed to confer protection against HBV infection. The AAAG haplotypes was observed with higher Ficolin-2 levels and viral load. The significant distribution of variable Ficolin-2 serum levels observed across HBV patient groups imply that Ficolin-2 may play an important role in innate immunity against HBV infection and of disease prognosis. However, further studies are required to elucidate and reconfirm these interactions between HBV and Ficolin-2 proteins in the disease outcome.
